# The power of the group: How neuroscience supports expanding the therapeutic dyad through group psychotherapy

**DOI:** 10.3389/fpsyg.2025.1631855

**Published:** 2026-01-15

**Authors:** Patrice Duquette

**Affiliations:** Private Practitioner, Birmingham, MI, United States

**Keywords:** active inference, Bayesian inference, epistemic trust, group psychotherapy, mentalization, mentalization of interoception, psychotherapy, psychodynamic psychotherapy

## Abstract

Group psychotherapy represents a therapeutic modality that provides unique affordances for patients, allowing layers of engagement by the patient in the psychotherapeutic process that are not available in dyadic psychotherapeutic treatment. This article considers psychodynamic group psychotherapy through the lens of current neuroscientific concepts. In doing so, we offer a framework for clinicians to consider how beliefs and habits that are originally adaptive become maladaptive and persist, and why patients hold on to maladaptive beliefs about the world and their own agency that are not reflective of the present-day moment. Neuroscientific proposals describing how early-life adaptations may result in longstanding false inferences regarding cause and effect in the world and within their emotional experiences will be evaluated alongside related psychological concepts. We will present and define the importance of neuroscientific concepts to the practicing psychotherapist, such as predictive processing, active inference, and the mentalization of interoception, as applicable to psychological and behavioral processes evidenced by the patient. The value that group psychotherapy specifically adds to processes needed to generate meaningful change will be explored in depth. How the milieu of group psychotherapy presents epistemic affordances to the patients in the group, which can then be leveraged in multiple ways to increase therapeutic benefit, will be addressed. Affordances in the group that offer patients unique interpersonal opportunities to address intrapersonal experiences such as mentalization of interoception and the activation of inference processes, creating lasting change, will be examined. The means by which interactions among group members decrease epistemic vigilance, increase epistemic trust, and facilitate epistemic foraging, thereby supporting necessary changes in mentalization capacity for patients, will be described. A case example will bring to light the elements, both neuroscientifically based and psychologically based, that are presented regarding the power of the group. Through access to and engagement with the process opportunities the group presents, patients can be supported by both therapists and other group members, affecting meaningful changes and ongoing present-moment adaptations over time for the individuals.

## Introduction

Clinicians involved in the practice of psychotherapy attempt to help patients sort out perceptions, actions, cognitions, and concurrent embodied emotional reactions that reflect necessary adaptations for the individual in their past but are no longer fitting for the present moment. These early adaptations generally occur as the patient tries to resolve uncertainties in their relationships and internal and external environments, often at times when they were not developmentally equipped with the means to do so effectively. Actions that achieved some degree of success in resolving uncertainty are typically repeated, along with any ensuing perceptions or cognitions. Over time, this repetition leads to habitual patterns taking precedence in how the patient experiences themselves and their world. The experiential and relational disconnect between the past origins of these patterns of behavior and the present day often induces a crisis in the patient’s life, leading to despair.

In this article, we will first consider current neuroscientific concepts that explain how these implicit, habitual responses take hold in the brain and why they persist beyond the disturbances that induced them. This requires an overview of the requisite constructs at a depth that is meant to support the psychotherapist’s understanding of the content and processes they entail. After consideration of psychological constructs that are considered necessary to promote change through psychotherapeutic treatment, the neuroscientific and psychological perspectives will be brought together. Group psychotherapy will be considered in depth regarding the specific resources and provisions it provides to address long-held, and likely false, beliefs; creating tools that will enable a change in current experience, as evidenced through perception, action, cognition, and emotional regulation. While the focus is on group psychotherapy as therapeutic treatment, this article provides an explication of underlying processes that touch on a wider range of considerations regarding process orientation and interventions for any clinician.

## Neuroscience concepts

We start with basic neuroscientific concepts that are necessary to understand key elements of change in therapeutic relationships and how the addition of group psychotherapy to dyadic relationships increases the potential for change in patients.

### Interoception, homeostasis and allostasis

The process wherein the nervous system “senses, interprets, and integrates” neural and humoral signals—originating within all tissues of the body, providing a “moment-by-moment mapping of the body’s internal landscape across conscious and unconscious levels”—is termed *interoception* (Khalsa et al., 2018). Interoceptive signals flow to the brain through designated neural fibers, engendering the perception of pain, temperature, hunger, thirst, muscle ache, itch, sensual touch, visceral urgency, or nausea, among many other sensations (Craig, 2008). Interoceptive sensations may be subpersonal (pre-reflective), may be accessible to consciousness, or may remain unconscious. Interoception serves essential roles in physiological processes such as homeostasis and allostasis and also subserves experiential processes such as emotion.

Homeostasis maintains essential physiological stability within the organism, keeping the body within close range of set points amidst stressors in the internal and external environment that could cause the organism to deviate from viable physiological regimes (Cannon, 1932). Interoceptive sensations can be experienced by an individual as pleasant or unpleasant, which induces motivation within the individual to act to limit immediate exposure to unpleasant sensations or seek out pleasant sensations, either unconsciously or consciously (Craig, 2015). Such motivated behaviors precipitated by interoceptive signals are necessary to maintain homeostasis and effectively guide homeostatic corrections through allostasis (Craig, 2002; Strigo and Craig, 2016; Critchley and Garfinkel, 2017; Stephan et al., 2016; Sterling, 2014).

Allostasis utilizes interoceptive information to prospectively preserve the stability of the organism when homeostasis is challenged, maintaining “stability through change” (Sterling, 2014) regarding the body’s biological needs. Allostatic mechanisms are instantiated through behavior, autonomic nervous system (ANS) activity, or initiating changes in the internal bodily environment. Interoceptive sensations play a role in allostasis as motivational signals inducing control over body states to decrease the effects of physiological perturbations (Sterling, 2012, 2014; Stephan et al., 2016; Petzschner et al., 2017). Perturbations that allostasis must address arise not only from the immediate physical needs of the individual but also from their social environment (Stephan et al., 2016).

The motivation resulting from homeostatic and allostatic processes is necessary to preserve energy and protect bodily integrity, leading to emotional inference through these embodied processes (Duquette and Ainley, 2019). Motivation is fundamental to emotion: motivation stimulates a change in action readiness that serves to maintain or change one’s position relative to a stimulus (Frijda, 2007), reflecting a valuation of the meaning of a stimulus to the body (LeDoux, 2002), or serving as a necessary mapping of the rewarding or punishing aspects of different stimuli onto the action system, thereby supporting subsequent approach or avoidance (Rolls, 1999). Emotion occurs through such means, instigated within the milieu of bodily states and mechanisms, and as such cannot be “controlled” as common parlance insists; what can be controlled are the behavioral responses to emotion. Emotions that the patient can express through mental actions that categorize and contextualize such experiences as feelings (Damasio, 1999) allow an important point of entry for the patient in psychotherapy, furthering exploration and insight regarding how emotions are stimulated by current bodily-based experiences and the residue of experiences from other times and relationships.

Emotional awareness necessarily follows from the evaluation of these motivational processes expressed as emotion and experienced as affect, evidenced through changes in arousal, action tendencies, and related cognitive processes that are either unconscious or conscious. Smith et al. (2020) propose a definition of emotional awareness as conceptual categorization and a process orientation. They argue that the semantics of emotion is an expression of underlying affect, with this language reflecting changes in affect over time, and that this categorization process affects goal-directed interaction with cognition. As noted above, encouraging our patients to pause and elaborate on emotional experiences, allowing deeper exploration of the sensations emanating from their bodies, encourages the development of emotional awareness.

### Inference in the brain

We next consider how predictive or inferential processing in the brain—which is constantly attempting to discover the cause of sensations from the inner and outer environment—is the basis for perception, cognition, and behavior.

Because the causes of sensation in the environment are hidden from the brain (i.e., the brain cannot step out and know the state of the world), the causes of sensation from our internal or external environment can only be determined through inferring what could be producing them. The brain thus serves as an “engine of prediction” (Schoeller et al., 2021), constantly attempting to infer the cause of sensation through hypotheses about states of the world. The concept of “predictive processing” offers a way to understand and engage this inferential account. The brain generates hypotheses, or “generative models” (Clark, 2016), of what could be causing sensations at any given point in time, necessarily to make sense of the internal (body) and external (world) environment. Such generative models anchor all physiological processes, perception, action, and ultimately cognition.

### Predictive processing

In the predictive processing account, inference processes rest on “priors” (expectations, beliefs). These serve as initial guesses about what in the internal or external (physical or social) environment could be causing sensations. Thus begins a “predictive” process of iterative testing of the sensation against the prior, diminishing any discrepancy through “prediction error,” which expresses the difference between what is sensed and the prediction of those sensations under prior expectations. This prediction error is then used to drive neuronal representations that resolve prediction errors, thereby updating prior beliefs into posterior beliefs, i.e., new expectations that are informed by sensory evidence. This process is known in statistics as Bayesian belief updating. The end result of this process is termed a posterior expectation or belief that reflects a change in the prior (Clark, 2016).

### Bayesian inference

Expanding upon predictive processing, Bayesian inference is a statistical formulation of inference processes in the brain that formalizes predictive processing, reflecting how beliefs can be optimally updated using new data (Parr et al., 2018). The brain is proposed to function as a “Bayesian observer,” constantly processing incoming sensations to infer the most likely causes of sensations within the internal and external environment. Perceptual inference can therefore be understood as an attempt to maximize the evidence for a generative model regarding the causes of sensory data. This follows because the prediction error, or surprise, is inversely related to model evidence. Frith states that the importance of Bayes’ theorem resides in how it “provides a very precise measure of how much a new piece of evidence should make us change our ideas about the world” (Frith, 2007).

The brain’s generative model is considered to have a hierarchically structured architecture (Friston, 2010), with neuronal activity reflecting iterative Bayesian inference occurring up and down the hierarchy. Sensory information enters at the lowest level and is matched against predictions (under a hierarchical generative model). Successive prediction errors constitute an informational signal as we move up the hierarchy, updating priors at each level within the hierarchy. This results in a picture of precision-weighted prediction errors ascending from one level to the next, generated by descending predictions from higher levels to lower levels—and ultimately the sensory levels.

Precision (salience or reliability) is a very important element of Bayesian inference. More precise prediction errors will have greater weight in the updating process and a greater effect on the outcome (posterior). Precision weighting contextualizes the influence of sensory information through attentional processes. This contextualization requires a prediction of precision regarding the reliability of prediction errors at sensory and higher (i.e., prior) levels. If this reliability, or precision, is not predicted properly, false inference can ensue because the brain is attending to the wrong kind of information or ignoring important sensory cues that would otherwise induce belief updating.

If the higher-level belief has more precision, i.e., one is more confident about prior beliefs, it will have more influence on the lower-level representations and will dominate perceptual inference—and ensuing action selection. Such a process highlights the importance of precision regarding the tendency to false inference (Connolly, 2022). All psychopathologies could be considered to be improper belief updating, or false inference, which will be examined in greater detail later (Friston, 2022).

### Active inference

Bayesian belief updating is not static; rather, it is a continuous flow of sensory input that enters from the environment, and individuals constantly compare incoming sensations against predictions. As a precision-weighted prediction error arises, it can be resolved in different ways: there is an updating of the prior, termed perceptual inference, which changes the predictions to match sensations. Alternatively, one can act upon the world to sample sensations that match the prediction; this is active inference. Active inference can be implemented by physical action (e.g., looking somewhere else) or mental action (e.g., changing attentional focus), thereby changing predictions of precision and effectively reducing prediction errors by attenuating the precision (Parr et al., 2022). Active inference thus subsumes perceptual inference, as changes in perception occur through processes of mental action: by directing attention to increase the precision of a particular kind of sensory evidence or attenuating the precision in the sense of sensory attenuation (Friston, 2022).

Active inference entails priors over policies, with policies being a sequence of actions. The precision of priors over policies has much influence on action selection and decision-making at higher levels of the hierarchy (Smith et al., 2022). Imagine the forms of inference that occur when you are reading a book and want to write a note in the margin. The pencil is lying next to you on the arm of the chair; as you are so engrossed in the book, you reach for it without looking. The movement of your arm is generated through the prior beliefs that underwrite the intention to reach for the pencil, which are fulfilled through motor reflexes. As your hand touches the arm of the chair—and does not touch the pencil—perceptual inference realizes, “Oh, that is the arm of the chair, not the pencil,” signifying the need to discern where the pencil is through an active change in attention. You turn your head to locate where the pencil is in space, actively inferring, “Oh, there is the pencil,” and subsequently pick it up, make your note in the margin, and continue reading.

### Interoceptive inference

A special case of active inference, “interoceptive inference,” refers to hierarchical inference processing that includes interoceptive sensation (Gu and FitzGerald, 2014; Ondobaka et al., 2017; Owens et al., 2018; Allen and Tsakiris, 2019). Such inferential activity constantly monitors changes to bodily states in support of homeostasis and allostatic efforts to maintain physiological set points proactively, evaluating current interoceptive sensations against generative models or expectations. The result of this inference process is the contextualization of autonomic reflexes and reactions to cues in the body that are emotionally salient for our patients. Ultimately, this monitoring of changes related to body states—through interoceptive inference and subsequent necessary adjustments of homeostatic and allostatic processes—gives rise to emotional experience (Sterling, 2012; Quadt et al., 2018; Seth and Friston, 2016).

There is one exception to this movement of prediction errors upward, at the (sensory) bottom of the hierarchy, where the prediction errors can descend to the effector organs of the ANS and elicit autonomic motor reflexes (Quadt et al., 2022). This diversion to the ANS, which depends on attenuating the precision of ascending prediction errors, will become relevant when we address clinical phenomena. In brief, if the precision of ascending prediction errors is attenuated, the prediction errors will be resolved through motor autonomic reflexes via homeostatic processes. However, if the precision of ascending prediction errors is increased through selective attention, there will be consequent belief updating and, crucially, a suspension of homeostasis in favor of more deliberative allostasis that depends upon perceptual inference in the CNS.

### The free energy principle, precision, and uncertainty

Bayesian inference represents the brain’s best efforts at decreasing uncertainty (by resolving precision-weighted prediction errors). The Free Energy Principle is a proposal that all living organisms act to limit uncertainty (i.e., average surprise or precision-weighted prediction errors) to remain in their characteristic states (Friston et al., 2006; Friston, 2010). It presents an elegant account of how inferential processes maintain homeostasis, which sustains self-organization and facilitates survival. Furthermore, as homeostatic and allostatic processes are elemental to survival, the Free Energy Principle presumes that activity regarding the self-regulating mechanisms of the body must be given the highest priority in inference processes (Allen and Tsakiris, 2019).

The full extent of this principle is beyond the scope of this article. Yet, it is important to recognize the impact the biological imperative to act to minimize uncertainty has on the development of false inferences from early life onward, which may persist with increasing precision. It is the formation and persistence of false inferences that produce the resulting psychological, physical, and emotional distress that our patients experience.

To illustrate the interpersonal imperative to act to minimize uncertainty, consider when uncertainty occurs in relationships without being resolved. Imagine being in a loud restaurant with a friend, trying to hear them amidst the noise. As they speak to you, you mistakenly infer they said one thing and respond. The reactive look on their face—as they try to comprehend your response—elicits a prediction error. However, if you do not pay attention to the facial expressions of your partner (i.e., this attention increases sensory precision to ensure another round of iterative testing), then you will fail to infer that you have misunderstood the discourse. And if, in turn, they do not take any further action regarding their inference about your response, the conversation will spiral into confusion and misunderstanding. If one of you then gets up and leaves the table, insisting to the other that “you have never understood me and will never understand me,” the relationship can degrade to the point of ending. This simple example highlights the importance of directed (joint) attention and actively sampling sensory information from the world to maximize the reliability of information and minimize uncertainty.

### Clinical implications regarding the mentalization of interoception

We will now distinguish between interoceptive inference as a pre-reflective, subpersonal (just out of awareness) assimilation of interoceptive bodily cues and personal, reflective attention to interoceptive cues that raise emotional feelings into awareness, which is the *mentalization of interoception* (Duquette and Ainley, 2019). In this view, emotions and feelings are essentially hypotheses or constructs that provide the best explanation for the myriad of interoceptive and exteroceptive cues, from the body and world, that have to be explained. The hypothesis “I am anxious” is a simple example of one possible explanation for a breadth of visual, auditory, somatosensory, and, crucially, interoceptive sensations.

### Mentalization of interoception

The term mentalization, as used here, is in line with other uses of the term, i.e., the intention and ability to understand mental states (Fonagy and Allison, 2014; Campbell et al., 2024). Mentalization of interoception, an “ongoing process of intentional, self-reflective evaluation of interoception” (Duquette and Ainley, 2019), offers a point of entrance for the patient to evaluate long-held beliefs and resulting emotional reactions that do not reflect the present moment and consider alternatives that are more reflective of the current moment and might better explain current sensations.

The basic premise of mentalizing interoception is that attending to interoceptive cues in a safe therapeutic setting allows patients to build a repertoire of alternative hypotheses about their emotional state, which they can then evaluate in relation to their original belief or hypothesis. This necessarily means elevating subpersonal emotional experience to a reflective level that, we suggest, offers the patient a chance to not only express feelings that are usually avoided as too painful or scary but also to consider whether these bodily feelings reflect the current psychotherapy setting. For this to occur, we propose that (i) the ability to mentalize interoception is necessary for therapeutic change, and (ii) this ability can only emerge if we consistently help the patient attend to bodily-based sensations in order to increase the precision of interoceptive sensations, providing a resource for deeper evaluation of the body’s responses to emotional reactions—that may underwrite false inferences based on early life adaptations—in the safety of the therapeutic relationship and setting (Duquette and Ainley, 2019).

To begin, we note that the therapist must first recognize that the patient is responding in ways that do not appear to reflect the present moment. This presumption includes the idea that the patient is responding as if they are explaining a harmless stimulus with a suboptimal prior, inherited from childhood, generating an emotional reaction (often outsized) that produces autonomic sequelae. The therapist draws attention to the bodily responses to the ANS sequelae (note that they cannot call attention to a prior, as it is generally subpersonal or unconscious), which often heighten the emotional reaction, either through strengthening the precision of the prior or the sensory prediction error. Through relational means, the therapist encourages the patient to stay engaged with them (and others if in a group) even as unpleasant sensations surge in the body and the feeling gains perceived veracity.

As the patient starts to recognize that the feelings and physical responses do not fit the present moment—i.e., the safe setting of the therapy room and relationship(s)—this will establish a new higher-level conscious prior that recognizes the autonomic reaction they are experiencing is not fitting to the present moment. As the prediction errors between the sensory data of the ANS reaction and the new higher-level prior interact, sensory precision is attenuated by the new prior (as it has gained increased precision weight relative to the prediction error), leading to a calmer state in the patient and reinstating corrective homeostatic mechanisms.

To engage the patient in this process of exploration that allows for the processing of experienced interoceptive sensations in the current moment and possible explanations for such reactions, the strength and resources of the therapeutic relationship are vital (Duquette and Ainley, 2019). They allow the patient to engage at a deeper level with the support they are receiving in the moment, to focus their attention on their body and express feelings, to experience an increase in discomfort and fear, and yet not quickly bring habitual (maladaptive) priors to the fore, thereby resolving their unattenuated interoceptive sensations. With this relationship at their back, the patient can then shift their attention to the present moment and assess whether these feelings are fitting. Such processes allow the new, higher-level prior an opportunity to “duke it out” with the prediction error, with the update leading to a calmer and more receptive state, which facilitates further change for the patient through ongoing practice. In other words, increasing the subjective awareness of the bodily experience and its relationship to emotion and feelings is necessary to revisit high-level prior beliefs and build a repertoire of alternative plausible emotional explanations for “what I am feeling.” This may lead to more authentic relational interactions with the therapist in the moment.

After such experiences, a patient can sometimes make an association to the past that clarifies why they react to the world in the way they do. They may then be able to detach those reactions from their behavioral processes because they have allowed the intense (precise) feelings to update, and possibly replace, the extant (historical) prior by subverting its precision relative to sensory evidence. Most therapists have the experience of encouraging the patient’s emotional experience while trying to find the language of feelings to describe such experiences, yet not allowing this to take the patient away from the experiential aspects of emotions, especially as these relate to the physical aspects of the experience. It is a fine line to keep the patient’s attention on the ongoing process in the moment while they are cognitively “researching” what related experiences or priors may also be active, and to prevent them from resorting to habitual behaviors or priors that derail such a constructive process.

The core tenet of this framework is that the body’s sensations furnish interoceptive evidence for a particular emotional state that can be recognized explicitly if, and only if, there are alternative emotional states of being from which to select. The historical priors will hold sway unless the patient is offered the opportunity to discover alternatives to living out the body’s habitual responses to feelings derived from those priors. Repeated practice, both within the relationship and outside of the therapeutic setting, gives the patient an increasing ability to generate self-calm and a thoughtful perspective in the face of perceived threat (Duquette and Ainley, 2019).

We now consider these neuroscientific principles in relation to their role in the therapeutic process of group psychotherapy. Starting with the development of mentalization and epistemic trust, we will then consider how affordances available in group psychotherapy support patients in improving such processes by diminishing the effect early adaptations have on their current experience. Ultimately, we will consider how the group supports the updating of priors through opportunities that encourage patients to be more self-observant, curious, and insightful, and improve their ability to mentalize interoception due to the increased number and quality of interactions within the group environment. A case example will illustrate specific elements.

## Psychological concepts

### Mentalization and epistemic trust

As described above, a central aspect of mentalizing interoception—in the context of psychotherapeutic intervention—is the act of mentalizing. In this act, the patient takes a subjective perspective on their bodily reactions, considering that these reactions may have several alternative explanations, and that the stimulus does not necessarily hold the meaning they initially inferred. With the therapist offering the patient an informational perspective that they are actually safe—amidst powerful, disorienting emotional experiences and bodily feelings—the patient is encouraged to continue such mentalization and thereby become skilled at recognizing and interpreting the causes of these sensations at a conscious or propositional level.

In continuing our consideration of mentalization in the psychotherapeutic process, we will first describe the importance of mentalization in early life dyadic interaction. We then consider how epistemic trust follows from a patient’s ability to engage in mentalization, contributing to their capability to remain present when they have strong feelings, even when their prior beliefs insist that they must avoid or flee from these feelings and the people who witness them.

### Origins of mentalization

Mentalization, as defined by Fonagy and Allison (2014), is the “capacity to understand others’ and one’s own behavior in terms of mental states.” In colloquial terms, mentalization is a process of imagining how behavior may result from thoughts, feelings, and intentions, whether one’s own behavior or that of another person. This requires a capacity to hold internal propositional perspectives regarding the origin and purpose of behaviors, which can subsequently be more or less available to individuals, given the variables at play—physically, emotionally, and relationally—at any given moment.

We begin with early infancy to consider how even the *possibility* of mentalization originates within the caretaking relationship. In the earliest period of infancy, the infant has no prior beliefs, beyond what Allen and Tsakiris (2019) label as the “first prior.” They propose that during this time, “the autopoietic principle at the basis of the FEP [Free Energy Principle] acts as a kind of ‘first prior’” (Autopoiesis is the concept that the system can self-organize to maintain its integrity; literally, to self-create, assemble, and maintain). They further assert that from birth, the interoceptive signals that instantiate homeostasis are afforded primacy amidst all other sensory inputs. This primacy continues throughout life to protect the body from life-threatening (i.e., surprising or high free energy) dyshomeostasis. With only primitive reflexes available to the baby and homeostatic processes creating internal sensations, the infant is left adrift in a bodily-experienced sea of a “*blooming, buzzing confusion*” (James, 1890).

As the infant’s body moves against the external world within caregiving relationships, these dyadic interactions—between infant and caretaker—generate sensations that gain purchase and are processed hierarchically, generating prediction errors that can begin creating alternative priors other than the “first priors” described above. This is proposed to occur through “embodied mentalization,” described by Fotopoulou and Tsakiris (2017) as a “dynamic” process of creating and updating generative models of what is causing the sensations in the infant’s body and about the external environment. This repeated sensory input, at the lowest hierarchical levels of the brain, supports early model building in the infant’s brain. As sensory organs such as the eyes come under more direct oculomotor control, the infant will (independently) seek out the caretaker’s eyes, and the consistent eye contact—and calming of bodily reactivity—acquires epistemic value for the infant, increasing the likelihood they will seek eye contact. This interaction is a “first step into someone else’s mental world” (Frith, 2007, p. 143), allowing a minimal differentiation between the infant and caretaker to occur, possibly instigating a slight sense that “I be.”

The caregiver’s mentalizing capacity scaffolds the developing child’s ongoing attempts at developing a sense of an “other” than themselves; the burgeoning sense of self and other supports successive leaps into mentalization; an early comprehension that the caregiver has intention toward them, experienced through the consistency of the caregiver’s actions; and, further along in development, that this “other” has feelings and thoughts. Acknowledging that these proposals involve many layers of supposition regarding possible experience in a non-verbal and non-purposeful infant, it is nonetheless recognized that interactions with caretakers facilitate the development of mentalization in the infant (Fotopoulou and Tsakiris, 2017), with the outcome highly dependent both on the caretaker’s responsivity and consistency with the infant and many other life circumstances present during this developmental time period.

### Epistemic trust

As therapists, early in the therapeutic engagement, we must consistently evaluate each patient’s potential for mentalization through their interaction with us and also the historical reading they relay of early life interactions. Over time, promoting the patient’s awareness of how they are imagining themselves and another while recognizing their limits in doing so encourages further development of their capacity for mentalization.

An outcome of being able to mentalize is epistemic trust, described as an individual’s level of willingness to consider information received from others (Campbell et al., 2024; Fisher et al., 2024; Fonagy and Allison, 2014). Krupnik also connects epistemic trust to the meaning of trust in active inference terms, which is how an individual “‘adopts’” another’s “generative model as reliable, thus establishing a shared model” (Krupnik, 2025. p. 7). In this way, epistemic trust is a measure of the trust an individual experiences regarding the information received from another, i.e., to what extent the informant is reliable, personally relevant, or generalizable (Campbell et al., 2024; Fisher et al., 2024; Fonagy and Allison, 2014; Bateman et al., 2021).

As the development process is fraught with interference, it is easy to imagine how readily an individual could encounter difficulty trusting that information offered by others is relevant to them and/or generalizable. The process of developing trust begins early in the relationship with caretakers. As a caretaker mentalizes the infant’s state, a sense of being recognized can be induced, along with the caretaker’s benevolent attitude and intentional stance. This benevolence includes a consistent recognition of the infant’s needs, relaying attentiveness and awareness back to the infant, and showing care for the infant, affirming that they remain safe amidst change. The experience of consistency and sensed benevolence can increase the potential for epistemic trust during important developmental stages and enhance the potential for meaningful engagement with information offered by significant individuals throughout life.

Cues from a person transmitting information, which include behavioral or verbal cues that they are intent on communicating something to another, are an important part of the relaying of information; Fonagy and Allison (2014) term this ostensive cueing. They further note that ostensive cueing encourages an attentional set in the receiver that important information is coming, that the information has personal relevance and is related to other social information that is also relevant, and so ought to be remembered and stored together, so that the content is retrievable together. The value of ostensive cues is that they signal implicitly to the other that “we must go beyond a specific physical experience and acquire information that will be relevant across a range of settings” (Fonagy and Allison, 2014, p. 374). Epistemic trust allows an individual to accept such cues and proceed as if the information they receive has value to them.

Another purpose of such cueing is to support the relaxation of epistemic vigilance. This vigilance occurs as an individual holds a position regarding the speaker that they may not have information that is applicable, or they are not benevolent in their intentions regarding the information (Sperber et al., 2010; Fonagy and Allison, 2014; Campbell et al., 2024). The value of a reliable, intentional, attentive, and soothing caretaker is evident here. If an attachment relationship has significant disturbances, then cues indicating that it is safe enough to relax epistemic vigilance will not be recognized, and both informant and information will be kept at a remove. This remove does not allow either the updating of the generative model of the informant or the acceptance and use of the information to assess its relevance and benefit for the infant.

Disturbances in epistemic trust are categorized as either epistemic mistrust, evidenced as caution due to a high possibility that the speaker’s motives and information may have harmful intent (Kumpasoğlu et al., 2025); epistemic credulity, or taking information in too readily with no consideration of the speaker’s credibility (Fisher et al., 2024); or epistemic freezing, whereby an individual only defends their existing knowledge base (or current generative model or policy) and is unwilling to address the other’s perspective, even if their own is patently not helpful in the moment (Connolly, 2022).

As knowledge gained through epistemic trust gains currency, the patient’s ability to experience the mental states of the other as having their interests in mind will increase, allowing them greater capacity to recognize ostensive cues from the other, consider the information relayed to them, and imagine what it means in relation to themselves. In the psychotherapeutic relationship, one of the most effective means to bring epistemic trust to an operational level—and decrease the impact of disturbances that diminish it—is by supporting, encouraging, and instigating mentalization in the patient through all available relational means. This does require that the therapist remain authentically involved and responsive to the patient, including earnestly considering the patient’s efforts at mentalizing the therapist’s perspectives, expectations, and intentions toward the patient. At the proper time, this can involve acknowledging whether the patient might have identified something the therapist was not aware of but recognized upon further consideration. Additionally, the therapist verbalizing an understanding regarding the patient’s expressed statements about what they imagine, even if the therapist does not find it to be accurate regarding themselves in the moment, both interactions explicitly and implicitly encourage the patient to continue the process of exploration and expression.

## Clinical considerations in group psychotherapy

As we now consider the affordances and opportunities that group psychotherapy offers, we will first briefly address the distinct differences between dyadic therapy and combined dyadic and group psychotherapy. We then consider in greater depth how interactions with members of the group (including the therapist) create opportunities for a more conscious and intentional delineation of possible underlying prior beliefs that were adaptive earlier in life but are no longer adaptive and require change. The group environment is distinctly different from the patient’s usual environment, and this change could make it less tenable for the patient to use prior beliefs and policies that they have maintained, up until that juncture, in their usual environments (Connolly, 2022). The quality of relationships in the group, which changes over time as the patients become more familiar with each other, offers an increasingly palpable felt sense of space and safety that the patient can utilize for themselves, opening up “epistemic channels” (Campbell et al., 2024) and allowing alternative information from sources they trust, which they can use to test their long-held prior beliefs. Real safety and a felt sense of safety are imperative if the patient is going to open up to the possibility that the way they believed the world to operate and what that meant for them was not accurate, because this entails an increase in uncertainty. Recognizing they are safe is the most important means to counter this sense of uncertainty.

In the author’s practice, patients are seen in psychodynamically oriented individual and group psychotherapy. The group meets at the same time each week; within the group, there are two co-therapists. The patient is also seen in weekly individual treatment with one or the other of the therapists. Within the first session or two of the individual patient’s evaluation, they are told that there will be a binding commitment expected of them and the therapist. This binding “commitment to not act on feelings” is described as committing to an intentional effort to consider whether an impetus for action is reality-oriented or stemming from an emotional reaction. The typical first response of patients is to say, “If I am not acting on my feelings, what am I acting on?” Expressing understanding for such a position, the therapist draws the patient’s attention to the likelihood that feelings do not necessarily have to have anything to do with reality, and thus behaviors precipitated only by feelings can more often lead to adverse consequences.

Through several exchanges within the evaluation period, the ability of the patient to keep the commitment is closely evaluated by the therapist. The patient is told that while it may not be immediately clear and comprehensible, this commitment is necessary for the work of therapy as it keeps the patient (and others around them) safe. In other words, the process of therapy allows deeper access to emotions that may have no association with the present, and old beliefs may have more access to behavioral expression. The commitment provides a necessary pause in behavior to evaluate the genesis of the impetus for action, especially with their therapist and the group. They are encouraged that—through the work of therapy, and subsequently throughout life—they will be learning the deeper meaning of the implications for living differently. Throughout treatment, the commitment is reiterated, explored, explained, and called upon to diminish not only clear episodes of “acting out” but also the more subtle behaviors and mental actions (such as strongly held prior beliefs and their related propositions) that are infused with emotion and lack a basis in reality.

The groups are process-oriented, with the time offered as a resource for all patients to use as they see fit. The group meets at the same time every week; patients are expected to be present regularly, and the patients expect that each other will keep the group apprised of when they will not be in group. A group comprises eight to ten patients. The patients in the group are not selected through any specific matching process regarding gender, age, or diagnosis.

While the original binding commitment extends to no physical contact between patients, with explicit permission, a therapist may offer touch as a means to help a patient secure a sense of safety, self-awareness, or encouragement for emotional expression. Again, this is done only with a direct question to ask the patient if they are willing, and an explicit answer of “yes.” At times, under the same constraints of explicit agreement, a patient may be offered the opportunity to physically move in a way that supports the elicitation, expression, and exposition of feeling states so that they may then consciously reflect on an otherwise hidden expression of emotion.

Such a process excludes psychosis as a diagnosis for this treatment, as the ability to reality test is essential. Aside from that, diagnoses can run the gamut. The most significant criteria are the ability of the patient to consistently put forth effort to comprehend and keep their binding commitment, attempt to separate thoughts from feeling states as best as they can, and participate in individual and group sessions in a consistent fashion.

While the particular therapeutic process depicted herein does have distinct qualities, the author maintains that the elements described throughout—regarding the therapeutic alliance, psychodynamic considerations, boundary management, patient commitments, and the address of transference/countertransference (or generative model expression)—are likely to be present in most psychotherapeutic styles. Practitioners are encouraged to consider combined group and individual treatments as powerful forms to engage with patients toward the goal of decreasing false inferences, as can be addressed through relational affordances that increase active inference possibilities, such as the mentalization of interoception.

## Group process and its provisions to the members of the group

Group psychotherapy offers unique epistemic (relating to knowledge or its validation) affordances (resources the environment offers) within the group process, which highlight the safety of the group environment and provide further means of support to the process of meaningful work to change long-held beliefs. The co-therapist relationship offers opportunities to split the transference, with one therapist providing a backdrop of emotional holding while the patient tries to untangle the beliefs and feelings that stimulate them to respond to the other therapist in ways that do not fit the moment. There are also unique relationships between individual group members and to the group as a whole, centered around shared agreement—to keep the commitment described above—to intentionally maintain an observant position regarding inner and relational processes to discern the impetus for perceptions, thoughts, and actions. This commitment is addressed whenever a new patient enters the group, along with the requirement for confidentiality, so that all members of the group know that each has made and will keep such commitments.

Within the layers of interaction within the group, the affordances that the group offers to patients can be categorized regarding (1) relational aspects, (2) epistemic affordances, and (3) generative processes.

### Relational affordances

In the group, a patient has an opportunity to see their individual therapist interact with other patients. Transference regarding the therapist—from a Bayesian perspective—involves the experience and expression of early generative models regarding caretaking others (amongst other models). Initially, observing the other patients’ encounters with the therapist will instantiate affective responses expressed as bodily reactions. Reflecting on such interactions over time will allow for the revision of earlier beliefs, reflected in changes in the affective response. The consistent observational perspective concerning the therapist in the group, along with the resulting epistemic value of the information to the patient—regarding the experiential outcome in the body—is distinctly different from the observational perspective the patient can take in individual sessions.

In the group, the increase in instances of interaction between the therapist and other patients offers the patient many more chances to assess if the therapist is consistent, authentic, and emotionally available with other people than in dyadic interactions. These qualities are descriptive of the real relationship, which is an important aspect of the therapeutic alliance, offering an experiential anchor for patients who may struggle with thinking and relating in the midst of strong feelings, allowing them to gain an observant position at those times, thereby facilitating the work of psychotherapy (Duquette, 2010).

Patients will see other patients in the group challenge the therapist in many ways; some patients will persistently test the investment of the therapist in them, others will persistently wait to see if the therapist will approach first, and others will try to take all the therapist’s time. As a patient observes others in the group, with the opportunity to see the therapist react consistently to other patients, this can relax epistemic vigilance, increasing their openness to receiving information from the therapist and evaluating it from an initial position that it holds meaning for them.

Opportunities for the patient to see the therapist as benevolent increase with each interaction of the therapist with the other members of the group. As the experience of benevolent intention is extended to other members of the group, epistemic vigilance can diminish. As patients observe different members express empathy toward each other and intercede in conversations when someone believes another patient needs more time and space to describe their emotional state or explain a problem they face, these interactions further lessen epistemic vigilance and uncertainty. This decreased vigilance supports an increasing sense of safety in the environment, especially as a patient may relate to content that others express, but most importantly in the process of the group, how it is managed by the therapist, and sometimes other patients.

### Epistemic affordances

As the patient observes others in the group, they gain traction in recognizing feeling states and their bodily expressions, furthering an observant intentional stance toward themselves amidst others. The manner of interactions that occur—and the way in which the therapists stay attuned to the process, supporting movement toward engagement with others and mentalization of the self and others—adds a layer of predictability to the process. This is an informational (epistemic) affordance that increases the experience and expectation of safety, increasing the likelihood that patients will engage in the process of the group, especially when their feeling state is most fraught, or their physical state is most fearful.

Epistemic affordances also come into play when patients are not directly involved in an interaction with another at a given moment. Their affective state is less charged, and they can pay more purposeful attention to other interactions, supporting mentalizing processes that allow the discerning of similarities with the other. They may recognize what the other patient is spontaneously feeling or what has happened with the therapists, which instigates a feeling reaction in them. Both are opportunities to explore their own experience, with the relational distance increasing their observational and mentalization capacity. This information can be useful in several ways to the observant patient, decreasing relational uncertainty, offering chances to explore how the similarities they recognize might reflect early adaptations that were required in their lives and the models that formed from that necessity, and providing an occasion to empathize with the other and offer themselves grace.

During such attentional periods, they can examine how those who are interacting are sharing information and for what purpose. As interactions unfold, they can attend to other patients’ emotional reactions, beliefs about the world, and responses to the therapist, and they will often compare these interactions to those that they themselves usually have in the world, from the perspective of both parties in the observed interactions. Paying attention to the patient involved in an interaction—and to how other members of the group are responding—expands their attentional capacity and also the intentional aspect of attention. As they allow themselves to remain observant, experience reactive states, and ultimately participate, this will signal a decrease in epistemic mistrust in the observing patient, which increases the available informational resources for the patient, including the valuable resources of other group members.

The therapists will be a central feature for interaction, but often other patients who have either similar emotional struggles, difficulty with elucidating their emotional state, or similarity in the behavioral expression of underlying models of themselves in the world will start or join in interactions with other patients. As the patients interact with joint attention to each other regarding what is being explored and attempt to consider how either might infer differently regarding the results of the original inference process, this mutual activity engages active inference as a socio-cultural process (Bouizegarene et al., 2024). These mutual inference interactions gain from the weekly consistency of contact, better allowing each to predict the other patient’s behavior and imagine how the other’s model might weight the current consideration and point that out, thereby bringing current states into better relief in the inference process and changing the outcome to reflect a more present moment one.

### Generative affordances

Patients will feel and express strong feelings in the group, be encouraged to discern their bodily reactions to emotions, and to imagine a label for feelings in the moment. Such language expressions can increase the emotional lexicon for others in the group. Group members may more intentionally reflect upon their observations of another patient, including their behaviors, the way they speak, and other “tells” that they have come to know in each other that indicate aspects of experience the other individual may not recognize due to strong feelings, pushing past their own discomfort to do so. They may speak to the other patient and offer their observations, inviting them to consider whether they have really explored other choices and that there may be other signs they can pay attention to, which may lead them to a different conclusion. The observing patient may reflect back to the other patient their insights related to experiencing their own reactive feelings, empathetic feelings, and their intuitions regarding the meaning behind the patient’s behaviors. In their approach, they are often intent on signaling that while they are reacting to aspects of the interaction and the patient, they are making efforts to clarify and separate their experience from that of the other patient. The therapist sometimes intervenes to “hold” one or the other patient in an observing posture to help them remain involved in the interaction with intentional purpose and self-awareness.

The observing patient may also intervene if the therapist has offered their perspective to the other patient, and the observer believes that the patient accepted the therapist’s position too readily. The patient involved in the interaction may be giving too much weight to the statements by the therapist and not enough credence to their own experience. The observer is promoting a more observational stance, effectively challenging the limits of the informational content the other patient contends they have received from the therapist, due to excessive epistemic credulity. Such support to question an authority figure would not likely have been available to the patient, and the support of the observant patient encourages a more thoughtful process for both.

These interactions signal an opening to further explicate their experience, with language and meaning that reflect the informational gains for both the individual involved in the interaction and the observer. This is a “cue-driven social-cognitive adaptation of mutual design,” which Fonagy and Allison (2014, p. 373) highlight as “ensuring a highly effective and efficient transfer of culturally relevant knowledge.” The observing patient is not merely an affordance offering “knowledge” in the form of content at that juncture but is also attending to the process in a way that encourages curiosity in each of them as they engage. These processes gain increasing relevance to the group as interactions between patients continue, thereby supporting the group’s cultural adaptation of the psychotherapeutic position, “process before content,” more effectively than if only driven by the therapist’s attempts to direct the process away from content-based interactions. When this position is supported by the therapist—and held by the group—there is an increased likelihood that curiosity and observational stances will generate active inference among the members of the group. This will also promote the co-creation of generative models among the group as a whole as a means to decrease uncertainty amidst the ongoing process of change in the individual members and within the group itself.

## Group resources that support active inference

### Epistemic affordances

As the members participate in the group, at any given time, one member may enact old models of themselves, i.e., insisting that the therapist does not “understand me” or “really see me” at a moment of strong feelings. Other members of the group will offer their memory of instances when the patient did, in fact, experience the therapist as earnest and understanding. The other patients may even be able to describe an exemplary interaction and cite the patient’s own words back to them that recognized the therapist as someone who can see them and appreciate their emotional experience. The patient will often look surprised and may even insist that it was a “different thing” and minimize the other’s proof. As their attention is further brought to bear on their assertion at that time, the therapist and others will invite them to seek other evidence in the moment to prove or disprove their stated belief. This evidence might be the sensation they are feeling in the moment that could be holding their attention and distorting their memory of the therapist’s stance toward them at another time. It might involve replaying the incident that they are stating proves they are right, paying attention to the origins of the incident and the timing of the feelings that occurred during the interaction. The group will continue to offer their perspectives, and the patient will be encouraged to consider how the information sources in the group are viewing the patient at this moment and see if they can sense the support as earnestly given, or if the others have an investment in the therapist only.

The patient is often limited in their behavioral repertoire during times of strong feelings and confusion when long-held beliefs become rigid; they do not readily look at others, respond more hesitantly, and do not attempt to recognize interactions offered by other patients as safe. They may be ruminating to themselves or nursing an emotional state they feel strongly about at that time. To help them attend to the present moment, they may be invited to sit on the edge of their chair, feel the ground under their feet purposefully, and begin to look around at the people in the room. If they quickly glance around, they will be encouraged to slow down and seek out eye contact with others (sometimes such recommendations are offered by other patients). This purposeful sensory focus on the physical sensations of the moment supports an increase in attentional resources, often eliciting a more exploratory position in the patient, furthering a deeper understanding of the intentions of others in the room.

Over time, the patient may slowly reconcile the overly strong emotional reaction they have to the therapist by reflecting on historical information they may have and the information they receive from the group. Other patients may remind them of times they were in a similar emotional state and held such convictions. In this engagement, the observant patient invites the patient to “epistemically forage” (Parr and Friston, 2017) and explore their sensory environment to seek information that could confirm or disconfirm their present model of themselves and the world. As they consider different perspectives, these interactions also have epistemic value for the patient and afford them the opportunity to affect change in their emotional position, which could help them proceed differently. The therapist remains purposeful in not pushing for a resolution to any stated beliefs; rather, they encourage the patient to “keep it open” and continue to probe the therapist and test their beliefs. Over time, this becomes an ostensive cue to the patient that their strong reaction is welcome in the room and valuable to them, for reasons not entirely clear in the moment but that may become clearer.

The members of the group are offering epistemic affordances to the patient in the midst of disorganized emotional states and disturbing beliefs through alternative perspectives and stated empathy for the “place” the other finds themselves in, amidst other interactions. The group as a whole also creates affordances, as the patient who is experiencing strong feelings pauses to attend to their sensory experience of the room and imagine the intentional set and safety of the group as a whole, while sitting with their emotional states amidst others who do not try to talk them out of such states due to their own needs, creating an anchoring presence amidst storms of strong feeling. While such efforts will not drastically change the generative model and fully disconfirm the prior at any given point in time, the activity within the group supports the patient in developing an increasing ability to mentalize regarding their interoceptive experiences and relational interactions, facilitating the testing of beliefs about policies they hold too tightly, creating essential change steadily over time.

### Value added by collective process

As the group process described above takes place, the individual patients are performing “precision estimates” regarding the reliability of the other patients’ opinions. Yon and Frith (2021) discuss how confidence in (i.e., precision of) certain beliefs is often estimated through metacognitive processes. These processes include an estimation of the uncertainty we have regarding our perception by accounting for just how reliable we consider this uncertainty estimation to be at a given point in time. If we sense that we are not very certain, this allows cognitive controls to engage processes that slow us down and prompt us to seek more information from others. When others share their own confidence in their estimations regarding beliefs they communicate to others, then “collective decisions can indeed become more reliable” (Yon and Frith, 2021, p. 9). While the group is not making decisions collectively, the responses and opinions they offer to others in the group facilitate a collective testing of hypotheses by the group.

Considering the example above—regarding the patient’s insistence that the therapist does not understand them—the group offered their opinions to support the patient in persisting with hypothesis testing. The patient was initially very certain and unwilling to allow for uncertainty. With other patients weighing in and reminding the patient of times they too were absolutely certain they were right, the patient had a chance to test the reliability of their certainty against what the others were saying. Observing the other patients also allowed them to gain sensory information to estimate how certain each person was about the interaction. Instead of continuing to engage adamantly with the therapist, they were offered the space to lessen the intensity, slow down the process, and engage more cognitive resources. These processes—regarding any experienced or stated belief that holds power—can take many directions, not at all neat and tidy, and can last over many groups. The most important actions of the therapist in the hypothesis-testing process are to remain attentive to the intensity of feeling of any member, continue to invite a review of present-moment sensory information, and allow the expression of other group members and those interactions to play out, providing more sources of information and perspective, which are all resources the patient can use to continue hypothesis testing.

### Benefits of social niche construction

As the patients learn the shared expectations of the group over time through their ongoing participation, the group becomes a socially constructed niche, uniquely for each patient (Bouizegarene et al., 2024). Within this niche, the participants in the group are the most important part, as they become a distinct representation of the world at large, with the affordances offered supporting new adaptive behavior in their local (group) niche (Bouizegarene et al., 2024; Veissière et al., 2019). Inference processes allow the patient to become a better modeler of this niche and its requirements, promoting better control over how they will interact within the dynamics of this system. Through active inference, the states of the individual patient and the group will become mutually predictable (Bruineberg et al., 2018; Friston, 2013). As the social aspects of the group become more predictable, the patient can “leverage” them as cultural affordances of the kind discussed above. The regularities of the group allow the different process aspects of the group to gain salience and support actions that are adaptive to the present moment. As fewer false inference processes occur, new actions will be met with improved outcomes, becoming the preferred actions and creating lasting change for the patient (Bouizegarene et al., 2024).

## Clinical case example

This case example from an interaction in a group highlights the many aspects of the group, outlined throughout this article, that provide affordances for the patient, thereby increasing mentalization about themselves and others. Interactions in the group offered a patient such affordances, ultimately allowing her to mentalize her interoceptive sensations, subsequently lessening the grip of feelings and altering her reliance on ineffectual priors that limited agentic behaviors. Elements of the description of the patient have been altered to maintain confidentiality.

These interactions occurred in psychotherapy group of eight patients; the therapist involved in the interaction is the author.

**Patient description**

Susan is a 35-year-old female, petite in stature, often self-effacing, not speaking spontaneously very often, but considered very insightful and caring by her fellow group members. When she did speak to them she was earnest, openly caring, and supporting them, especially at times of intense emotion.

Susan’s father left the family when she was young, and her mother remarried soon after. Her stepfather was extremely hostile to Susan, inflicting cruel physical punishments for things such as not doing the dishes or other household chores correctly. He could be suddenly physically abusive, such as suddenly striking out at Susan as he walked past. He insisted that she respond immediately to his every demand, and was a harsh critic who did not offer any help with schooling but would punish her for bad grades. Susan’s mother would not support her if she complained about what he did or his demanding nature. Susan remembers her saying, “just do what he says and he will stop yelling.”

Susan had difficulty with school, but was a polite student who asked few questions, so that she had to learn most information contextually. She left home soon after high school, having worked during high school to pay for anything that she wanted, with her parents providing only for basic needs. Susan sometimes worked two jobs at a time and was always frugal with her money. She was not married. While she did not avoid relationships with men, she also did not seek them out, waiting for a man who would be non-conflictual and kind.

She had worked at a small manufacturing company for the last 10 years and was the secretary to the man who owned the company. He appreciated her work but was not very direct about what he wanted, and Susan had to learn how to do much of the work on her own. He was also not available to her when she had concerns and expected Susan to manage various issues with others on her own, even if those issues had nothing to do with her position. She found this frustrating and anxiety-provoking, certain that she would do something to displease him. Other employees considered her competent and affable, a good resource, and managing the issues brought to her very well.

Susan became competent at her job but continued to have significant anxiety about even the smallest of her decisions or actions. She was certain she would do something wrong and be yelled at by her boss, others would become upset and angry with her, or she would be fired.

In her therapy, Susan ruminated often on small things at work or interactions that she thought would lead to bad consequences. She would have a strong sense of dread in the morning after ruminating as she fell asleep. She was often encouraged by the group to speak up about issues she had, or not to take on responsibilities that were not hers. Group members often spoke to her about her friendly engagement and insightfulness in the group as evidence of her competence. She was often skeptical of such recommendations or compliments, reciting reasons as to why she could not do the things she wanted, and minimizing her interactions within the group with gestures of self-deprecation. This lessened slowly over time, and at the time of this group session Susan was not reporting many concerns about work.

**Group session**

Susan began talking early in the group. She described, in great detail, one of the workers at the company whom she reported was loud and unpredictable. This individual would often come into the office and demand to see the boss, becoming more and more angry and yelling at Susan as she tried to explain her boss’s absence. Susan would try to resolve the issues as best she could. After such encounters the person would walk away, but Susan often remained anxious. Susan would tell the boss what had happened and ask for guidance as to how to handle the worker’s questions and his angry behavior. However, the boss dismissed her concerns and only told Susan that whatever she had done or would do was just fine.

Susan reported that the day of this group session, the worker had come into the office again, loudly and persistently complaining about something and insisting that he see the boss. Susan told her the boss was out at a meeting. The worker became more visibly angry and stood in front of Susan, gesturing and leaning over her desk toward her while continuing to complain loudly. The female therapist spoke up at this point and asked Susan what she felt when the person became loud.

Susan: “I get scared, I do not know what is going to happen!”

The therapist asked if she had thought to ask this person to leave when they became loud and agitated.

Susan: “Leave, how could I ask them to leave, they work there!” (Susan’s body visibly tensed, her voice rose in pitch and volume).

Therapist: “I understand that, but your work responsibilities do not include allowing others to yell at you incessantly, do they? You could restate to them that the boss wasn’t there now, and would they please come back later. That is a polite interaction between co-workers, is not it?”

Susan: “But they might not leave, and they could get angrier, and then what would I do?”

Therapist: “Well, you could get up from your desk and go to the door and ask them to step out of the office and return to their area.”

Susan: “I would be too afraid to do that, I do not know how they would respond, I cannot do that.”

Therapist: “Would your boss mind?’

Susan: “No, I do not think so, he would understand. But I could not do that, I am just the secretary, I’m not the boss. They probably aren’t going to listen to me.”

Group member: “But it’s your space, where you work, they do not get to bully you!”

Susan: “Well, yes, it is, it is my space, but they were just standing there. And yelling! I just did not know what to do.” (Susan’s eyes widened and moistened, she looked back at the group member plaintively.)

Therapist: “Hmm… Are you willing to try something?”

(As noted above, if the author or their co-therapist decide that a physical action or intervention might help someone, either with the therapist, by themselves, or in the direction of someone else, they are always first asked directly if they “are willing to try something.” The therapists wait for a spoken yes or no answer before proceeding. It is not a common intervention, and the patient must answer with the word, yes, if they agree to it. Not yeah or ok, as that response merely indicates compliance, not agreement. This adds clarity to the fact that they also have the prerogative to not agree.)

Susan: “Yes, I am, what do you want me to do?”

Therapist: “You stay there. I will move.”

The group members shifted in their seats and remained attentive, watching Susan carefully, a few exchanging knowing glances across the room, slight smiles indicating recognition.

The therapist got out of her seat and moved rather quickly across the room to where Susan was sitting. She stood in front of Susan, essentially toe-to-toe and making eye contact, not smiling but not frowning.

Susan’s eyes widened; she frowned and looked frightened and confused.

The therapist then inclined over her slightly, leaning forward at the waist.

Susan leaned back further in her chair. Her eyes widened more. She was not looking at the therapist’s face.

“Why are you doing that?” she said quietly.

The therapist remained silent and inclined closer toward her, still neither smiling nor frowning, and continuing to make eye contact.

Susan looked more scared though she did look at the therapist’s face without making direct eye contact. She said, “What are you doing?” slightly louder, and her hands moved slightly forward and then back. The therapist stayed standing still and not talking, not changing her facial expression.

Then Susan’s face changed, she stopped frowning, and she made direct eye contact. She then moved her hands toward the therapist more purposefully as she said, “Step back. Step back and out of my space, please,” with a firm, loud, voice.

The therapist stepped back a few steps and stood still making eye contact for a long moment. She then went back to her seat, where she continued to look at Susan and waited for her to speak.

Susan remained silent but continued to make eye contact with the therapist, looking at her intensely.

The therapist asked her what she felt in her body at that moment.

Susan: “I do not know, I can barely feel it, I cannot feel my legs. I feel like I want to cry. I did not like that feeling at all. I was so surprised and scared. I know you would not hurt me, and everyone was quiet so they did not think so, they would have had to have gotten you away if they thought so. It felt like you were just going to keep on coming and I could not do anything to stop you. I just wanted you to move away, get you away from me!”

Therapist: “But you did stop me, you told me to leave your space. Can you describe now what you were experiencing as you moved from leaning back to then moving your hands toward me and telling me to step out of your space?”

Susan: “I did not say it, not at first. You kept on leaning over, and you would not answer me! I knew I did not want you to stay there and I began to move my arms to push you, but that did not seem right, to push you. But I really wanted to push you hard and far away from me. The feeling of needing to get you away from me was strong. Then those words came to me and I could feel my arms start moving and I just said them.”

The therapist stated that Susan’s voice had been firm, and her hands had moved from behind her toward the therapist very directly, as she told the therapist to move away. The therapist said she could very well understand why Susan would have wanted to push her even farther away. Susan quickly said, “I would never have hurt you.” The therapist said she had no concerns Susan would harm her and then explained that she had been waiting until Susan could find the voice in her that she expected Susan had available. She sensed that if she waited, Susan could find the means to take the space that was hers, even though she saw Susan was very scared. She reminded Susan that she had seen her make her way through her feelings and expectations at other times and was confident that she had the means to do it then.

Susan then said again, “I did not like that, I do not like that feeling. I did not like not knowing what to do.”

The co-therapist quickly replied, “You surely found out what to do pretty quickly! With that voice anyone would move out of your space.”

Susan repeated, “I did not like doing it though.”

The therapist then said to Susan, “You keep on insisting that you did not like it, but you can recognize that you wanted to just push me away — hard. Your arms signaled you had power and that power could be used to protect yourself, and you did not believe you were powerless, at that moment certainly.”

Susan considered this for an extended period; the group was attentive to her and waited quietly. “I could never do that in his house, I could not even make a face without getting hit. I had to just keep quiet and let it happen and she never made him stop, even when I begged her! That’s why I left the house so quickly. But I know you will not hurt me, and that there must have been some reason [why] you were doing what you were doing. I was so sure that something bad had to happen, then it came to me, just say get away!”

Therapist: “So, you can fully claim your space and tell someone to leave you alone, you found your voice and told me to get out of your space. You knew it was your space, your body know it could move and you could protect yourself.”

Several members of the group spoke up quickly. One said to Susan, “Oh, you sure figured it out what to do, only two tries. And that voice, you found the voice I had heard before quickly.” Another affirmed, “I saw your eyes change and saw you figure out you could tell her to move, I knew you could do it.” A third group member remarked, “I was actually saying “back off” so loudly in my own head, and she was actually over there in your space, not mine, but it upset me over here and I was across the room.”

The group then spent much of the remainder of the session talking about how they were glad Susan had participated and that she had surely found an ability to claim her space, and did not have to continue “living in that old place full of feelings” but saw different ways she displayed her strength and the group was a safe place to express it so she could remember it in the future.

In the next group session, a male patient told the group about a very difficult time he had experienced some years ago, when he was verbally and physically harassed by a kid in high school over a long period and he did not know how to tell the person to leave them alone. He did not believe that he had had the prerogative to do so, and believed that if he had said something, others would have made fun of him. He was tearful as he spoke of this and said he was surprised to be talking about it. He had not spoken to anyone about those events and how he felt about them ever before, realizing how much shame he felt as he talked. The group addressed him directly about how difficult it must have been and offered support to help the patient stay emotionally available. The patient did connect his current willingness to speak of feeling so “small” to witnessing Susan’s efforts at not living the way that she “felt she had to,” and how she had discovered that there were other options available to her in how she saw herself and how she behaved.

## Discussion of clinical case

In this snapshot of the clinical process within the group, the patient begins with verbal ruminations about many elements of her workplace that leave her upset, feeling powerless, and very scared. Susan’s choice of words regarding her interactions at work carried the weight of her long-held priors from childhood. Her expression of her boss’s stance proved that people left her or were not actively supportive or protective (her deserting father and ineffectual, non-protective mother), and the description of how her co-worker expressed anger (and seeming dominance) reflected that it was “safest” to submit to others’ hostile behaviors rather than protest or remove herself from a situation (the abusive stepfather). At this juncture, Susan’s ruminations were gaining purchase as an affective inference, with the certainty of her subpersonal priors of threat creating an increasing sense of uncertainty regarding her safety, thus setting her body into high alert. The resulting stimulation of autonomic nervous system reflexes reflects the movement of prediction errors moving downward from the bottom of the hierarchy (rather than the usual upward movement), instigating the autonomic sequelae evidenced in Susan’s vocal constriction, the increasing muscle tension in her body, and her physical movement away from the therapist.

The therapist’s questions were intended to help Susan redirect her attentional focus toward her present-day abilities (and away from the perceived threat), pointing out actions she could take or support she could seek from her boss. The current lack of mentalization that Susan was evidencing indicated that further cognitive, language-based interactions were not interrupting her ruminative focus—only increasing the precision of experienced threat. The therapist recognized the necessity of activating inference processes to lessen the hold that fear had on Susan’s bodily experience, severely limiting an exploration of alternative hypotheses regarding another’s intentions and new, non-habitual behavioral responses. Realizing that Susan had many experiences with her over time during which she had felt safe and had tested the authenticity of the therapist’s commitment to supporting her efforts to change, the therapist decided that a physical intervention could be helpful. Such an interaction would offer Susan a relational affordance, actively supporting an increase in attention toward the relational environment, likely lessening her epistemic vigilance and increasing attentional control.

The therapist’s original intention was to simply stand in front of Susan, but as she approached Susan, she noted that Susan avoided eye contact, moved back in her seat, and remained otherwise passive. The therapist then decided to intrude in her physical space more purposefully, expecting that this would prompt a more activated response. The therapist did not intend to speak; rather, she simply wanted to see how Susan responded.

At first, Susan could only submit to the therapist’s intrusion, moving back in her chair, folding her body inward, and trying to limit the increasing anxiety she felt. As the therapist inclined toward her, Susan’s hands moved slightly forward and then back, and her demeanor began to change. As she reported later, she sensed that the therapist would not hurt her, and she reassured herself with this information and the recognition that the group would come to her aid if she needed them. She imagined pushing the therapist away as the therapist leaned closer, with a sense that she would forcefully move her “far away” to stop the intrusion. As she proceeded to speak in a louder voice, her questioning shifted from the general first question of “why” to the more specific, “What are you doing?” regarding the therapist’s behavior.

Susan’s behaviors and her process of imagining alternatives rather than just accepting the therapist’s intrusion are evidence of her beginning to mentalize her interoception, i.e., explicitly recognizing the causes of her autonomic arousal and how it could be remediated through volitional action. This remediation resulted from the movement of her arms and imagining moving the therapist out of her space. This countered prior convictions that she would be hurt in this kind of scenario and allowed her to begin imagining alternatives (initially that she could push the therapist “hard”). The deepening exploration of interoceptive sensation resulting from the “practicing” of an alternative model of herself, as she moved her arms and sought eye contact, supported a further attentional shift away from her ruminations and toward the present moment relationship with the therapist. The increase in interoceptive sensory stimuli from her body heightened the precision of interoceptive sensations and attenuated the relative precision of the historical prior, affording Susan an exploration of alternative explanations or new priors. The imagined “hard” push was discarded as she reached a realization that such action did not fit the moment. A new conscious prior arose, imagined and then asserted through her loud demand that the therapist move out of her space, along with firm movements of her arms to mark out the space. As her interoceptive experience verified this prior—she could assert her prerogative to stop an intrusion without reprisal from the therapist—this new conscious prior began to explain away and diminish the prediction errors that had been stimulating the autonomic reaction, facilitating a decrease in arousal.

The therapist inviting Susan to participate with her, expecting an explicit statement of agreement, was intended to lessen the strength of the current maladaptive feeling state, with the relational affordances offered to her eliciting a shift to a feeling state more reflective of present moment involvement. Such an intervention is in line with a process described in emotion-focused therapy, i.e., “activating more adaptive emotional states” (Pascual-Leone and Greenberg, 2020). The intervention did encourage a shift from fear and anxiety to an emotional response of boundary-setting anger, verbally directed at the therapist. The commitment to not act on feelings anchored this interchange, as Susan recognized an action orientation as non-viable; however, such an imagined response set in motion alternatives to choose from, resulting in her statement insisting the therapist move away. The value of the group relationships is signified in Susan’s statement afterward that she knew the group would intervene and help her if she were really in danger. Pascual-Leone and Greenberg (2020) contend that the extent of meaning-making that occurs in aroused states reflects “emotional productivity” and can “distinguish” a good outcome for a patient. Susan’s progression toward more adaptive behavior and fitting emotional reactions, recognizing the effect of her long-held priors on her reactions when she felt she had to submit, reflects the “emotional productivity” that Pascual-Leone and Greenberg (2020) assert is important for continued progression. A fellow group member’s observation of witnessing the physical expression of Susan’s renewed activated inference process during the interaction captures her meaning-making; they described seeing a change in her eyes that reflected to them she was then able to “figure out” that she *could* take action to stop the intrusion. This description has ongoing epistemic value to Susan as a reminder that volitional control of her attention is essential in times of fear, further increasing the likelihood that she will continue to progress toward meaningful change.

Susan spontaneously told the group about the interaction at work, verbalizing the ruminative thought process she had been engaged in all day by herself, indicating she believed the group could offer her epistemic affordances she did not have alone. The socially constructed niche of the group offered a safe space for her to express herself aloud, even as she felt her vulnerability more acutely, and that her group members would earnestly engage with her and offer alternatives that could help her. Within this new socio-cultural niche, Susan could test out different adaptive behaviors that she could not in her family of origin. She also navigated her initial hesitation about claiming her role in the process, telling the group how that would not have been possible in her family home. In the next group, another patient “leveraged” their experience of observing Susan move past her habitual model and shared a long-held shameful experience. He took action to expose this experience after seeing the results of Susan’s efforts, decreasing the uncertainty of what to expect as he allowed himself to be vulnerable, reflecting an inferential process that predicted the group would be supportive of him even when others had not been during his earlier family interactions.

The real relationship that had been developing between Susan and the therapist prior to these interactions became an important resource for Susan. The consistency of the therapist’s beneficence toward Susan, along with her authentic recognition of Susan enacting her prerogatives in their relationship, stood in sharp contrast to the fear response her generative model was currently stimulating. This aspect of the therapeutic alliance was the backdrop against which Susan could weigh her decision to participate with the therapist. Often, it is the real relationship that anchors the patient as they enter the group and the number of possible interactions expands, increasing the likelihood of maladaptive behaviors occurring.

Susan had been responding to questions up to the intervention, exhibiting epistemic trust in the process and the relationship as she continued trying to consider the questions. The therapist’s manner and voice inflections as she addressed Susan cannot be adequately described here, yet Susan’s stated agreement to engage with the therapist in an unknown activity reflects that she was responding to ostensive cues from the therapist. The question had been asked—and answered before—as they both knew and had witnessed in other patient interactions by Susan. This allowed Susan to relax her epistemic vigilance enough to open the door to another form of engagement, even though she did not know what it would be.

As the group members spoke to Susan at the end, they offered epistemic affordances to her, exclaiming that they were aware of the difference in her voice as she took her space and applauding her for that, encouraging more effort in that direction and reminding her they believed she could continue to change. She benefited from her group members witnessing her ability to manage the intensity of her emotions while expressing her prerogative—a very different experience from what she had encountered growing up in her family.

## Conclusion

Drawing from a neuroscientific perspective, all psychopathology is false inference; furthermore, the basis of the errors in inference is proposed to be due to “aberrant precision control” (Hobson and Friston, 2021, p. 19). Successful outcomes for the patient in psychotherapy therefore require enabling change—in this process of false inference—by countering the precision of priors, especially among the high-level prior beliefs that are considered fundamental to a model of self. These prior beliefs are slow to change, and aspects of the environment, such as relationships, play a major role in supporting their maintenance (Connolly, 2022).

Most early models of self—that are constituted by these high-level priors—begin in states of fear, due to the inherent sense of danger amidst uncertainty in the environment of infancy. Ongoing or recurring periods of uncertainty and unpredictability are also all too likely in important relationships, which could reinforce the precision of the early prior. Many times, each party in a relationship is too highly dependent on false inferences guiding their way through the difficulties of the relationship, unfortunately leaving the more vulnerable party open to the vagaries of guessing how to proceed. Yet, it is also true that being in a relationship is an absolute necessity for us to survive infancy and to continue developing. Psychodynamic psychotherapy, grounded in the consistency of the real relationship, serves as fertile ground to engage in relational interactions that can effect profound change in the distortions that have become draped over the patient’s generative models and greatly disturb the ways in which they see themselves and live in the world. It is imperative that the therapist continually evaluate the extent to which patients experience epistemic trust and build upon that with consistent, authentic, and meaningful engagement to support their efforts at increasing their capacity for mentalization.

We have proposed that group psychotherapy provides necessary relational interactions of a form that affords the patient more avenues and opportunities to activate inference processes in the midst of uncertainty and fear. Moving beyond the dyadic psychotherapeutic relationship, the multiplicity of relationships within the group offers opportunities to test and retest beliefs and their reality basis, and also—as the members remain committed to consistent, thoughtful participation—allows the development of a stronger relational basis from which each can participate in the process of psychotherapy. The relational, epistemic, and generative affordances that are available can powerfully increase the necessary felt sense of safety, allowing a greater number of relational interactions, increasing and deepening epistemic trust and lessening epistemic mistrust and epistemic vigilance within those relationships. This is vital as patients try to remain openly vulnerable during periods of challenges to their self-model, courageously activating inference processes anew. This requires relaxation of long-held priors that have been fundamental to the patient’s ongoing experience. Activating inference within the psychodynamic group process is encouraged through epistemic foraging, deploying attentional resources through the interactions in the group, and providing epistemic and relational support for the testing and retesting of maladaptive beliefs. This offers each patient in the group opportunities for testing beliefs and engaging in behaviors that more accurately reflect the reality of the present moment. Meaningful change requires persistent effort on the part of the patients and the authentic and consistent presence of the therapist, offering recognition of how difficult the process is, signaling when there is progress, and supporting hope for further change.

## Data Availability

The data is in a case study regarding one person and is not available due to ethical concerns.

## References

[ref1] Allen M. Tsakiris M. (2019). “The body as first prior: interoceptive predictive processing and the primacy of self- models” in The interoceptive mind. eds. Tsakiris M. De Preester H. (Oxford, United Kingdom: Oxford University Press), 27–45.

[ref2] Bateman A. Campbell C. Fonagy P. (2021). Rupture and repair in mentalization-based group psychotherapy. Int Jour Group Psychotherapy. 71, 371–392. doi: 10.1080/00207284.2020.1847655, 38449133

[ref3] Bouizegarene N. Ramstead M. J. D. Constant A. Friston K. J. Kirmayer L. J. (2024). Narrative as active inference: an integrative account of cognitive and social functions in adaptation. Front. Psychol. 15:1345480. doi: 10.3389/fpsyg.2024.1345480, 38903472 PMC11188712

[ref4] Bruineberg J. Kiverstein J. Rietveld E. (2018). The anticipating brain is not a scientist: the free-energy principle from an ecological-enactive perspective. Synthese 195, 2417–2444. doi: 10.1007/s11229-016-1239-1231, 30996493 PMC6438652

[ref5] Campbell C. Kumpasoğlu G. B. Fonagy P. (2024). Mentalizing, epistemic trust, and the active ingredients of psychotherapy. Psychodynamic Psychiatry. 52, 435–451. doi: 10.1521/pdps.2024.52.4.435, 39679701

[ref6] Cannon W. B. (1932). The wisdom of the body. New York, NY: W.W. Norton & Company.

[ref7] Clark A. (2016). Surfing uncertainty: Prediction, action, and the embodied mind. Oxford: Oxford University Press.

[ref8] Connolly P. (2022). Instability and uncertainty are critical for psychotherapy: how the therapeutic Alliance opens us up. Front. Psychol. 12:784295. doi: 10.3389/fpsyg.2021.784295, 35069367 PMC8777103

[ref9] Craig A. D. (2002). How do you feel? Interoception: the sense of the physiological condition of the body. Nat. Rev. Neurosci. 3, 655–666. doi: 10.1038/nrn894, 12154366

[ref10] Craig A. D. (2008). “Interoception and emotion: a neuroanatomical perspective” in Handbook of emotions. eds. Lewis M. Haviland-Jones J. Feldman Barrett L.. 3rd ed (New York, NY: Guilford Press), 272–290.

[ref11] Craig A. D. (2015). How do you feel? An interoceptive moment with your neurobiological self. Oxford: Princeton University Press.

[ref12] Critchley H. D. Garfinkel S. N. (2017). Interoception and emotion. Curr. Opin. Psychol. 17, 7–14. doi: 10.1016/j.copsyc.2017.04.020, 28950976

[ref13] Damasio A. (1999). The feeling of what happens: Body and emotion in the making of consciousness. New York, NY: Harcourt.

[ref14] Duquette P. (2010). Reality matters: attachment, the real relationship, and change in psychotherapy. Am. J. Psychother. 64, 127–151. doi: 10.1176/appi.psychotherapy.2010.64.2.127, 20617787

[ref15] Duquette P. Ainley V. (2019). Working with the predictable life of patients: the importance of “Mentalizing Interoception” to meaningful change in psychotherapy. Front. Psychol. 10:2173. doi: 10.3389/fpsyg.2019.02173, 31607993 PMC6774393

[ref16] Fisher S. Fonagy P. Zilcha-Mano S. (2024). More than meets the "I": a panoramic view of epistemic Trust in Psychotherapy. Psychopathology 58, 80–93. doi: 10.1159/000541667, 39467536 PMC11965823

[ref17] Fonagy P. Allison E. (2014). The role of mentalizing and epistemic trust in the therapeutic relationship. Psychotherapy (Chic.) 51, 372–380. doi: 10.1037/a0036505, 24773092

[ref9001] Gu X. FitzGerald T. (2014). Interoceptive Inference: homeostasis and decision-making. Trends Cogn. Sci. 18, 269–270. doi: 10.1016/j.tics.2014.02.001 24582825

[ref18] Fotopoulou A. Tsakiris M. (2017). Mentalizing homeostasis: the social origins of interoceptive inference. Neuropsychoanalysis 19, 3–28. doi: 10.1080/15294145.2017.1294031

[ref19] Frijda N. H. (2007). The Laws of emotion. Mahwah, NJ: Lawrence Erlbaum Associates.

[ref20] Friston K. J. (2010). The free-energy principle: a unified brain theory? Nat. Rev. Neurosci. 11, 127–138. doi: 10.1038/nrn2787, 20068583

[ref21] Friston K. J. (2013). Life as we know it. J. R. Soc. Interface 10:20130475. doi: 10.1098/rsif.2013.0475, 23825119 PMC3730701

[ref22] Friston K. (2022). Computational psychiatry: from synapses to sentience. Mol. Psychiatry 28, 256–268. doi: 10.1038/s41380-022-01743-z, 36056173 PMC7614021

[ref23] Friston K. J. Kilner J. Harrison L. (2006). A free energy principle for the brain. J. Physiol. 100, 70–87. doi: 10.1016/j.jphysparis.2006.10.001, 17097864

[ref24] Frith C. (2007). Making up the mind: How the brain creates our mental world. Hoboken, NJ: Wiley & Sons, 2007.

[ref25] Hobson J. A., Gott J. A. Friston K. J. (2021). Minds and Brains, Sleep and Psychiatry. Psychiatr Res Clin Pract. 3, 12–28. doi: 10.1176/appi.prcp.20200023 35174319 PMC8834904

[ref26] James W. (1890). The principles of psychology. New York, NY: Holt.

[ref27] Khalsa S. Adolphs R. Cameron O. Critchley H. Davenport P. W. P. W. Feinstein J. S. . (2018). Interoception and mental health: a roadmap. Biol. Psychiatry Cogn. Neurosci. Neuroimaging 3, 496–498. doi: 10.1016/j.bpsc.2018.04.007 29884281 PMC6054486

[ref28] Krupnik V. (2025). The therapeutic alliance and resistance as active inference: the role of trust and self-effficacy. J. Contemp. Psychother., (in press) 53, 207–215. doi: 10.1007/s10879-022-09576-1, 41467166

[ref29] Kumpasoğlu G. B. Saunders R. Campbell C. Nolte T. Montague R. Pilling S. . (2025). Mentalizing, epistemic trust and interpersonal problems in emotion regulation: a sequential path analysis across common mental health disorders and community control samples. J. Affect. Disord. 372, 502–511. doi: 10.1016/j.jad.2024.12.050, 39694336

[ref30] LeDoux J. (2002). Synaptic self: How our brains become who we are. New York, NY: Viking.

[ref9003] Ondobaka S. Kilner J. Friston K. J. (2017). The role of interoceptive inference in theory of mind. Brain Cogn. 112, 64–68. doi: 10.1016/j.bandc.2015.08.002 26275633 PMC5312780

[ref9004] Owens A. P. Allen M. Ondobaka S. Friston K. (2018). Interoceptive inference: from computational neuroscience to clinic. Neurosci. Biobehav. 90, 174–183. doi: 10.1016/j.neubiorev.2018.04.017 29694845

[ref31] Parr T. Friston K. J. (2017). Uncertainty, epistemics and active inference. J. R. Soc. Interface 14:20170376. doi: 10.1098/rsif.2017.0376, 29167370 PMC5721148

[ref32] Parr T. Pezzulo G. Friston K. J. (2022). Active inference: The free energy principle in mind, brain, and behavior. Boston, MA; London, England: MIT Press.

[ref33] Parr T. Rees G. Friston K. J. (2018). Computational neuropsychology and Bayesian inference. Front. Hum. Neurosci. 12:61. doi: 10.3389/fnhum.2018.00061, 29527157 PMC5829460

[ref34] Pascual-Leone A. Greenberg L. (2020). “Emotion-focused therapy: integrating neuroscience and practice” in Neuroscience of enduring change: Implications for psychotherapy. ed. Lane R. D. (New York, NY: Oxford University Press), 215–244.

[ref35] Petzschner H. Weber L. Gard T. Stephan K. E. (2017). Computational psychosomatics and computational psychiatry: toward a joint framework for differential diagnosis. Biol. Psychiatry 82, 421–430. doi: 10.1016/j.biopsych.2017.05.012, 28619481

[ref36] Quadt L. Critchley H. D. Garfinkel S. (2018). The neurobiology of interoception in health and disease. Ann. N.Y. Acad. Sci. 1428, 112–128. doi: 10.1111/nyas.13915, 29974959

[ref37] Quadt L. Critchley H. Nagai Y. (2022). Cognition, emotion, and the central autonomic network. Auton Neurosci. 238:102948. doi: 10.1016/j.autneu.2022.102948 35149372

[ref38] Rolls E. T. (1999). The brain and emotion. Oxford: Oxford University Press.

[ref39] Schoeller F. Miller M. Salomon R. Friston K. J. (2021). Trust as extended control: human-machine interactions as active inference. Front. Syst. Neurosci. 15:93. doi: 10.3389/fnsys.2021.669810, 34720895 PMC8548360

[ref40] Seth A. K. Friston K. J. (2016). Active interoceptive inference and the emotional brain. Philos. Trans. R. Soc. B 371:20160007. doi: 10.1098/rstb.2016.0007 PMC5062097 28080966

[ref41] Smith R. Friston K. J. Whyte C. J. (2022). A step-by-step tutorial on active inference and its application to empirical data. J. Math. Psychol. 107:102632. doi: 10.1016/j.jmp.2021.102632, 35340847 PMC8956124

[ref42] Smith R. Steklis H. D. Steklis N. G. Weihs K. L. Lane R. D. (2020). The evolution and development of the uniquely human capacity for emotional awareness: a synthesis of comparative anatomical, cognitive, neurocomputational, and evolutionary psychological perspectives. Biol. Psychol. 154:107925. doi: 10.1016/j.biopsycho.2020.107925, 32610156

[ref43] Sperber D. Clement F. Heintz C. Mascaro O. Mercier H. Origgi G. . (2010). Epistemic vigilance. Mind Lang. 25, 359–393. doi: 10.1111/j.1468-0017.2010.01394.x

[ref44] Stephan K. E. Manjaly Z. Mathys C. Weber L. Paliwal S. Gard T. . (2016). Allostatic self-efficacy: a metacognitive theory of dyshomeostasis- induced fatigue and depression. Front. Hum. Neurosci. 10:550. doi: 10.3389/fnhuman.2016.00550 27895566 PMC5108808

[ref45] Sterling P. (2012). Allostasis: a model of predictive regulation. Physiol. Behav. 106, 5–15. doi: 10.1016/j.physbeh.2011.06.004, 21684297

[ref46] Sterling P. (2014). Homeostasis vs allostasis: implications for brain function and mental disorders. JAMA Psychiatry 71, 1192–1193. doi: 10.1001/jamapsychiatry.2014.1043, 25103620

[ref47] Strigo I. A. Craig A. D. (2016). Interoception, homeostatic emotions and sympathovagal balance. Philos. Trans. R. Soc. Lond. Ser. B Biol. Sci. 371:20160010. doi: 10.1098/rstb.2016.0010, 28080968 PMC5062099

[ref48] Veissière S. P. L. Constant A. Ramstead M. J. D. Friston K. J. Kirmayer L. J. (2019). Thinking through other minds: a variational approach to cognition and culture. Behav. Brain Sci. 43:e90. doi: 10.1017/S0140525X19001213, 31142395

[ref49] Yon D. Frith C. D. (2021). Precision and the Bayesian brain. Curr. Biol. 31, R1026–R1032. doi: 10.1016/j.cub.2021.07.044, 34520708

